# Annual rhythm in immune functions of blood leucocytes in an ophidian, *Natrix piscator*

**DOI:** 10.1038/s41598-024-63033-8

**Published:** 2024-05-28

**Authors:** Alka Singh, Ramesh Singh, Arti Parganiha, Manish Kumar Tripathi

**Affiliations:** 1 Department of Zoology, School of Studies of Life Sciences, Guru Ghasidas Vishwavidyalaya (A Central University), Bilaspur, Chhattisgarh 495009 India; 2 Department of Zoology, Udai Pratap Autonomous College, Varanasi, Uttar Pradesh 221002 India; 3 School of Studies in Life Science, Pandit Ravishankar Shukla University, Raipur, Chhattisgarh 492010 India

**Keywords:** Annual rhythm, Immunity, Leucocytes, Lymphocyte proliferation, Seasonality, Immunology, Physiology, Zoology

## Abstract

Annual variations in animal’s physiological functions are an essential strategy to deal with seasonal challenges which also vary according to the time of year. Information regarding annual adaptations in the immune-competence to cope with seasonal stressors in reptiles is scarce. The present research plan was designed to analyze the presence of circannual immune rhythms in defense responses of the leucocytes in an ophidian, *Natrix piscator*. Peripheral blood leucocytes were obtained, counted, and superoxide anion production, neutrophil phagocytosis, and nitrite release were tested to assess the innate immune functions. Peripheral blood lymphocytes were separated by centrifugation (utilizing density gradient) and the cell proliferation was measured. The Cosinor rhythmometry disclosed the presence of significant annual rhythms in the number of leucocytes, superoxide anion production, nitric oxide production, and proliferation of stimulated lymphocytes. The authors found that respiratory burst activity and proliferative responses of lymphocytes were crucial immune responses that showed the annual rhythm. It was summarized that the immune function of the *N. piscator* is a labile attribute that makes the animal competent to cope with the seasonal stressor by adjustment in the potency of response.

## Introduction

Seasonal changes, imposed by the environment, are an inherent part of the life of animals, reflecting an interaction and coordination between individual internal rhythm and the environment. Annual variations in the physiological functions, including immune responses, have been deciphered in various vertebrate species^1–4^. The amount of time an organism receives light in a 24-h period is referred to as photoperiodism. Light provides sequential information for reproductive success at the time when the survival of offspring is optimum^5^. Along the line of these studies, investigations regarding seasonal fluctuation in immune functions have mainly been worked out in mammals. It has been observed that reduced photoperiod elevates cell count, lymphoid organ mass and immunoglobulins in mammals^6–8^. A notion suggests that elevated melatonin during winter is responsible for hyper-immune status during short days^9^. Freeman et al.^10^ and Wen et al.^11^ have suggested that most physiological processes, including immune responses, are melatonin-dependent. Among homeotherms, apart from mammals, a few reports are available in birds, where an alteration in defense mechanism has been attributed to changing photoperiods^12,13^. So far non-mammals are concerned, a few studies on the modulation of immune surveillance by blood cells have been performed in fish where significant annual rhythm has been reported in many immune parameters^14,15^. In an earlier study, we found circadian and seasonal alterations in nine immune parameters of splenic macrophages in *N. piscator*^16^. Reptiles are crucial heterotherms because of their significant phylogenic position and *N. piscator* is a potent contender for studies regarding vertebrate seasonality. There are two major lines of immunological investigations that have been considered in heterotherms. On one hand, the immune system is subjected to modification by environmental variables^17^, whereas on the other hand seasonal variation of the disease incidence can be the potential candidate for the death of the organism^1^. Migration, which is essential for reproductive success, has been shown to align with the annual predictable changes. Wingfield^18^ has proposed that *Saxicola torquata* show migratory behavior in captivity using external cue (day length). It has been suggested that the susceptibility of fish to pathogens is a season-dependent phenomenon^19–21^.

Leucocytes are the pivotal immune cells in the defense processes of organisms^22^. The circulating blood cells are the vital elements of the defense repertoire showing annual and daily rhythms. Twenty-four-hour rhythm in eosinophil count has been found in a reptile^23^, but no significant study was carried out on the possibility of the occurrence of annual rhythms in the immune functions of reptiles. In a previous study, we found circadian and seasonal fluctuations in the immune functions of cells from the spleen in an ophidian *Natrix piscator*^24^. Similarly, Munoz and Fuente^25^ have explained the seasonality in the leucocyte functions in the turtle *Mauremys capsica*, however, leucocyte immune rhythms in *N. piscator* remain largely unexplored. There are a few reports in the non-mammals where it has been investigated that immune responses are a function of seasonal alterations. Researchers of various literatures have found that the leucocyte immune functions are affected by annual variations. The leucocyte count is high in summer^26,27^ but oxidative stress of phagocytes is elevated in winter^28^. Literature review throughout the vertebrate group reveals that annual variations in immune status is a species-specific trait that seeks the opportunity to adjust through the climatic variables^29,30^

There is very limited literature available regarding season-dependent variation in immune molecules^31^. A few reports in the non-mammals suggest that physiological processes are affected by seasonal variation^1,17, 32^. In tropical and temperate seasonal breeders, such as reptiles, fluctuations in climate lead to the adaptation in physiological functioning to cope with the challenges^33^. The checkered freshwater keelback (*Natrix piscator*) is an oviparous seasonally breeding reptile found in many parts of Asia. It breeds from September to December when its gonads are maximal in size^34^. The chronobiological studies in reptiles’ immunity are a very undercovered area. The development of a systemic strategy to explore the annual alterations of immune functions across the vertebrate groups will be guided by the studies in reptiles. The class reptilia is a crucial taxon from the comparative point of view as reptiles seem to have sturdy innate immune responses that can be measured. The present research plan is attractive in how factors such as seasonal stressors affect the variations in immune functions. Many reptiles are long-lived and tolerate many pathogens^35^. Thus, an investigation of the immune responses in reptiles will provide an understanding of the evolution of this physiological response in other vertebrate groups. Looking at the above facts, this research was designed to explore the annual rhythmicity in blood leucocyte immune indices in *N. piscator.* The blood leucocytes were segregated from the whole blood and the innate immune responses were measured. Annual fluctuation in cell-mediated immune functions was assessed by analyzing mitogen-induced proliferation of lymphocytes.

## Results

**Figure 1:**
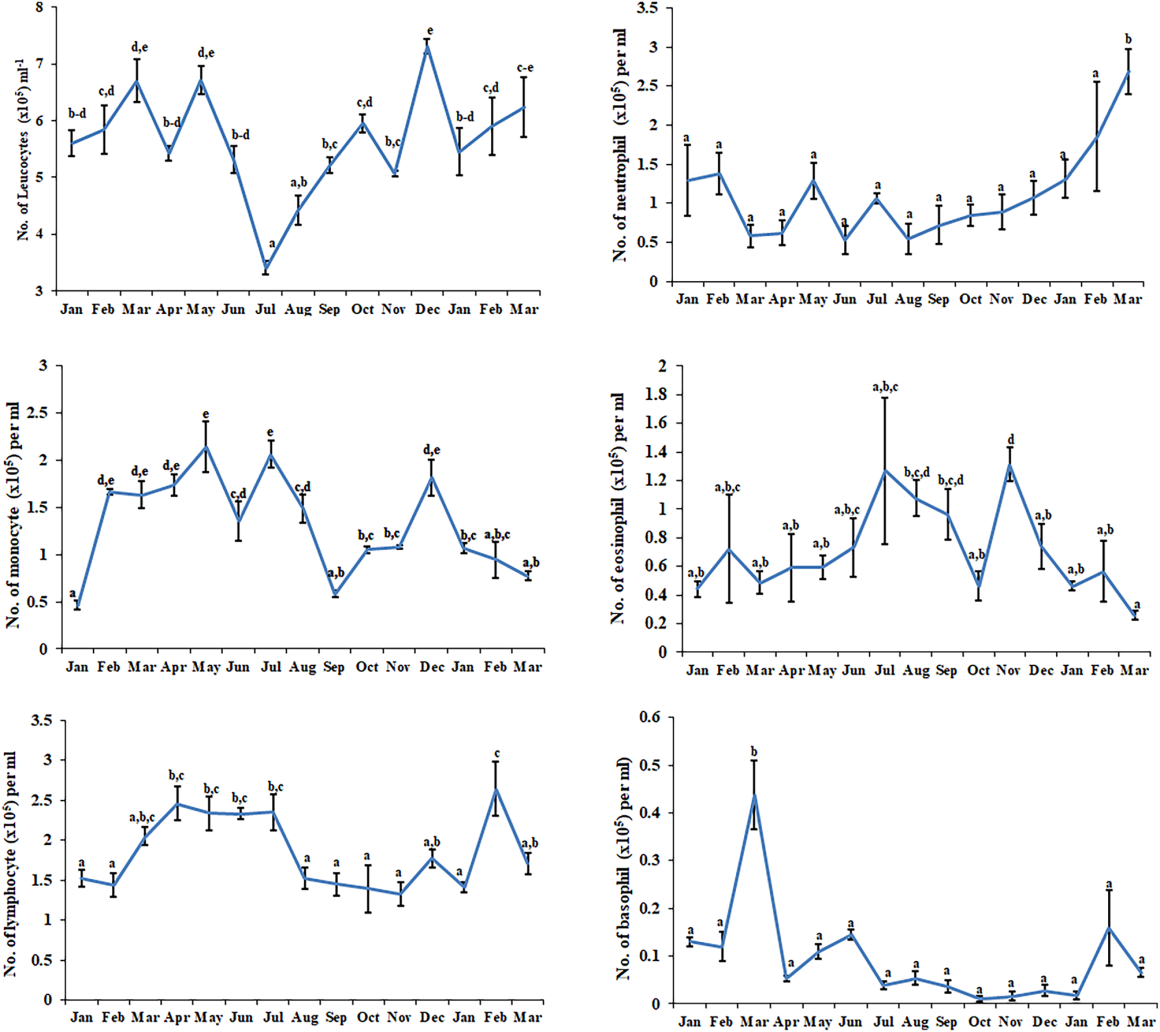
Seasonal differences in total and differential leucocyte count in *N. piscator*. Data were analyzed by ANOVA. The error bars having the same letter do not differ significantly (Post-hoc comparisons were made using Newman–Keul’s multiple-range test).

**Figure 2:**
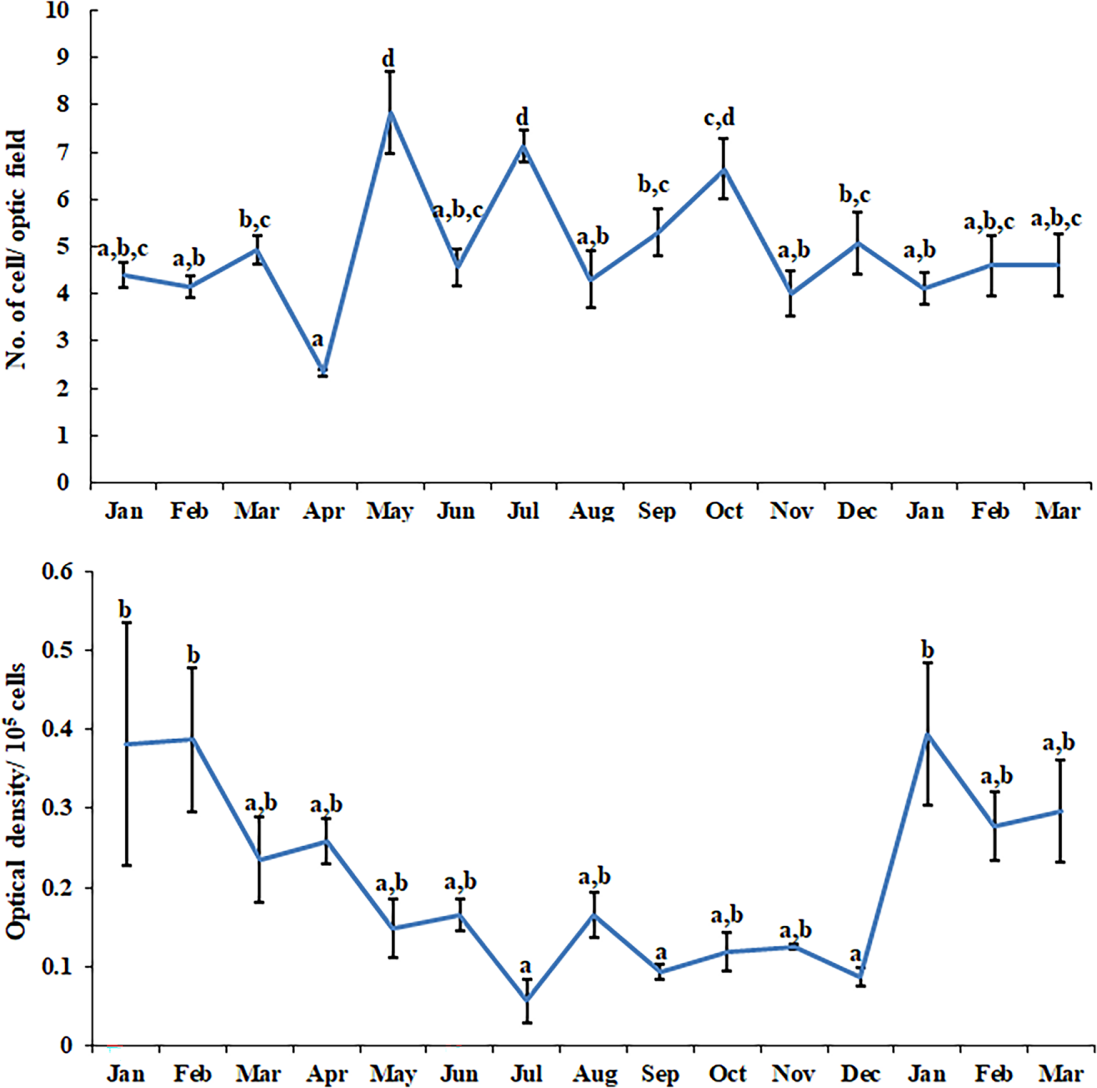
NBT slide assay (right panel)-The respiratory burst activity of leucocytes was determined as the reduction of NBT. NBT assay (left panel)-quantitative respiratory burst activity of leucocytes was determined as the reduction of NBT (Post-hoc comparisons were made using Newman–Keul’s multiple-range test).

The characteristics of annual rhythm (τ = 365.25 days) and six-monthly rhythm (τ = 182.625 days) in immune functions of leucocytes are shown in Tables 1 and 2 respectively. The total and differential leucocyte count varied significantly in the seasonal study (Fig. 1). Statistical analysis revealed significant annual rhythms in the total number of leucocytes during 12 months (df = 60; F = 9.990 and p < 0.001). Individual blood cells also showed significant rhythm as monocytes were maximum in May and July (df = 60; F = 10.950 and p < 0.001) but lymphocyte peak was obtained in February (df = 60; F = 3.567 and p < 0.001). Additionally, the annual data of neutrophil and basophil also resulted in significant changes with a degree of freedom of 60 each and F-value of 7.328 and 9.299 respectively with p < 0.001. Cosinor study revealed significant annual rhythms in the number of different leucocytes during 12 months. Superoxide anion production by leucocytes was assessed by NBT slide assay and NBT reduction assay. NBT slide assay and quantitative NBT reduction assay during different months are shown in Fig. 2. A statistically significant annual rhythm (τ= 365.25 days) was validated when quantitative NBT reduction assay and NBT slide assay were studied (df = 60; F = 3.671 and p < 0.001 and df = 60; F = 7.268 and p < 0.05 respectively).

**Table 1:** Characteristics of circannual rhythm (τ = 365.25 days) in immune functions of leucocytes.

Variable	Data points^a^	Rhythm detection^b^	Mesor (M ± SE)	Amplitude (A, 95% CL)	Acrophase (Ø, 95% CL)
Total leucocyte count	75	< 0.001	5.50 ± 0.12	0.75 (0.36, 1.13)	43.86 (9.66, 78.06) 4 February (1 January, 10 March)
Monocyte	75	< 0.001	1345.60 ± 61.14	406.98 (181.94, 632.01)	154.85 (122.74, 186.96) 27 May (25 April, 29 June)
Lymphocyte	75	< 0.001	1848.11 ± 56.10	520.83 (312.87, 728.78)	136.14 (114.96, 157.31) 8 May (17 April, 30 May)
Neutrophil	75	0.002	1037.39 ± 89.86	433.59 (136.03, 731.14)	51.38 (5.12, 97.64) 12 February (27 December, 30 March)
Eosinophil	75	< 0.001	752.16 ± 43.11	294.48 (150.46, 438.49)	243.35 (212.44, 274.26) 25 August (25 July, 25 September)
Basophil	75	< 0.001	86.49 ± 12.77	83.32 (37.74, 128.90)	99.79 (69.21, 130.37) 1 April (1 March, 2 May)
NBT	75	< 0.001	0.19 ± 0.02	0.12 (0.06, 0.17)	54.71 (24.52, 84.90) 16 February (16 January, 17 March)
NBT assay	75	0.016	5.07 ± 0.19	0.75 (0.12, 1.38)	217.20 (155.16, 279.25) 29 July (27 May, 30 September)
Percentage phagocytosis	75	0.121	57.14 ± 1.35	3.92	186.40 28 June
Phagocytic index	75	0.108	2.21 ± 0.07	0.22	321.63 12 November
Nitrite	75	0.170	53.77 ± 5.46	15.63	39.30 31 January
S.I.—Con A	75	0.003	1.41 ± 0.84	0.43 (0.13, 0.73)	173.33 (126.70, 219.97) 15 June (29 April, 1 August)
S.I.—LPS	75	0.018	1.51 ± 0.11	0.43 (0.06, 0.81)	87.89 (32.59, 143.19) 20 March (24 January, 15 May)

^a^Number of data points; ^b^rejection of the null amplitude hypothesis, significance of rhythm; M: rhythm-adjusted average; A: half of the difference between the highest and the lowest values in the fitted Cosine function; Ø: peak in degrees as a time lag with reference to the midnight of the 22nd December of the previous year; also expressed in month and day. Single Cosinor rhythmometry was employed to obtain the rhythm parameters.

**Table 2:** Characteristics of six-monthly rhythm (τ = 182.625 days) in immune functions of leucocytes.

Variable	Data points^a^	Rhythm detection^b^	Mesor (M ± SE)	Amplitude A, 95% CL)	Acrophase (Ø, 95% CL)
Total leucocyte count	75	0.108	5.68 ± 0.13	0.38	242.43 23 February
Monocyte	75	0.031	1350.54 ± 65.50	248.16 (18.20, 478.11)	311.89 (244.76, 379.01) 2 November (26 August, 10 January)
Lymphocyte	75	0.557	1858.65 ± 68.57	104.77	317.99 10 May
Neutrophil	75	0.103	1073.51 ± 93.96	283.37	95.42 24 September
Eosinophil	75	0.458	710.14 ± 49.40	87.51	328.94 21 May
Basophil	75	0.086	90.49 ± 13.93	44.10	137.47 5 November
NBT	75	0.015	0.20 ± 0.02	0.08 (0.01, 0.14)	88.13 ( 31.26, 144.99) 20 March (23 January, 17 May)
NBT Assay	75	0.382	4.98 ± 0.20	0.39	308.32 30 April
Percentage Phagocytosis	75	0.016	57.32 ± 1.30	5.35 (0.82, 9.88)	257.02 (198.76, 315.27) 8 September (11 July, 6 November)
Phagocytic Index	75	0.181	2.23 ± 0.07	0.18	258.18 10 March
Nitrite	75	< 0.001	56.17 ± 4.96	28.84 (11.15, 46.52)	173.85 (137.02, 210.67) 15 June (9 May, 23 July)
S.I.—Con A	75	< 0.001	1.40 ± 0.08	0.48 (0.20, 0.77)	226.29 (190.40, 262.18) 8 August (2 July, 13 September)
S.I.—LPS	75	0.250	1.55 ± 0.11	0.26	156.33 25 November

^a^Number of data points; ^b^rejection of the null amplitude hypothesis, significance of rhythm; M: rhythm-adjusted average; A: half of the difference between the highest and the lowest values in the fitted Cosine function; Ø: peak in degrees as a time lag with reference to the midnight of the 22nd December of the previous year; also expressed in month and day. Single Cosinor rhythmometry was employed to obtain the rhythm parameters.

**Figure 3:**
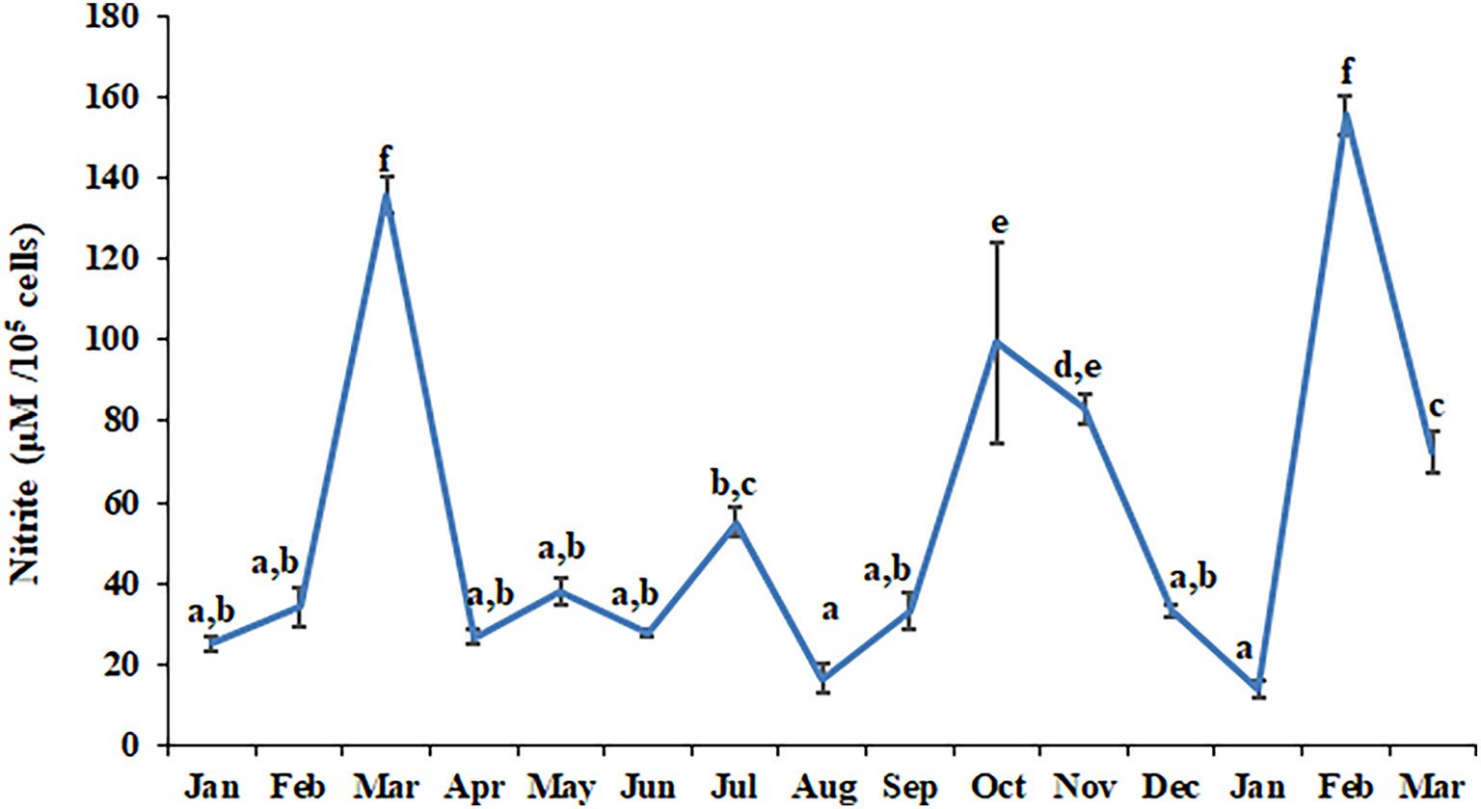
Nitrite release by blood immune cell in *N. piscator* during different months. The error bars bearing different letters differ significantly (Post-hoc comparisons were made using Newman–Keul’s multiple-range test).

**Figure 4:**
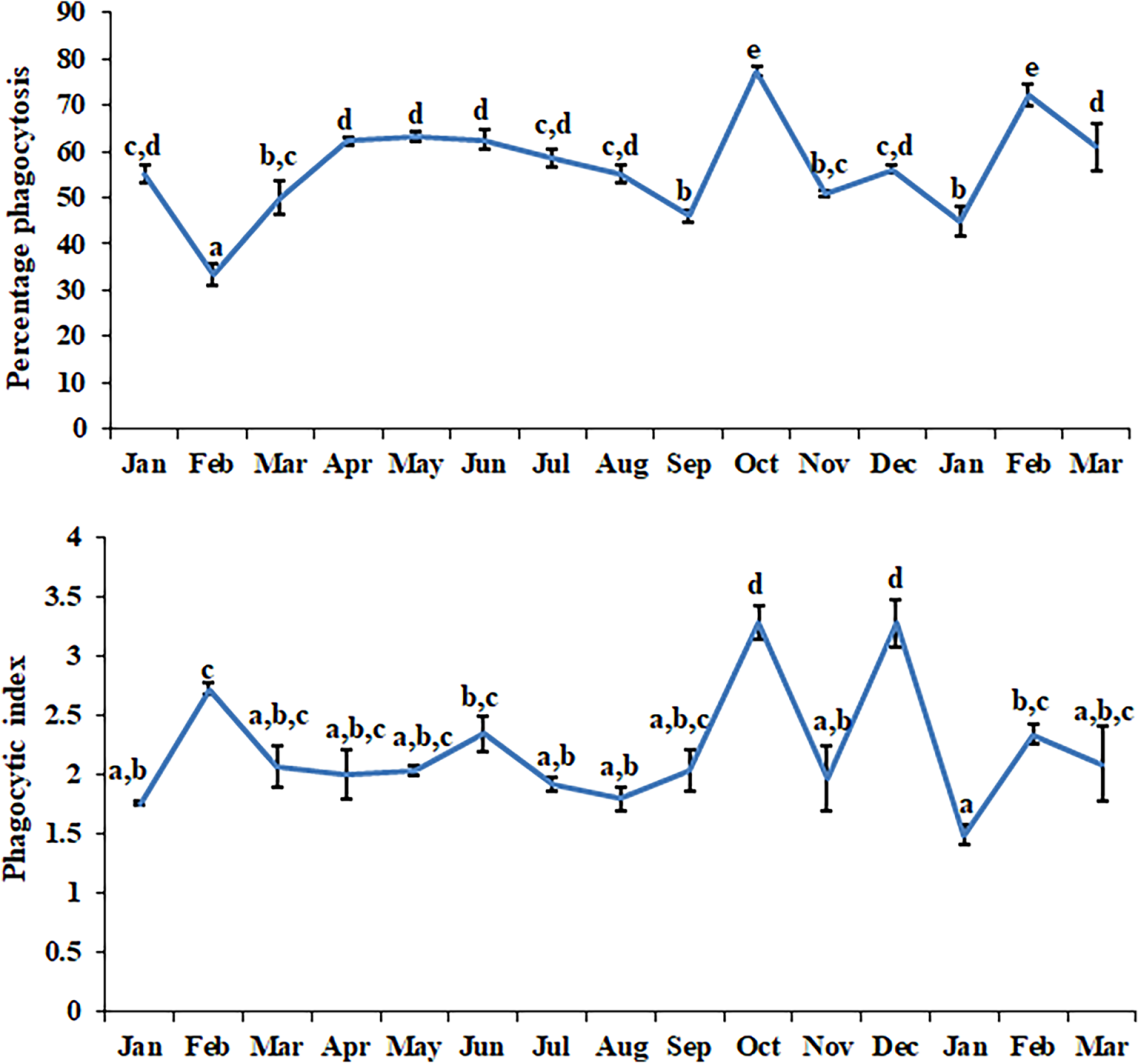
Annual changes in the percentage phagocytosis (PP) and phagocytic index (PI) by leucocytes in *N. piscator*. The error bars bearing the same superscript do not differ significantly (Newman–Keul’s multiple-range test, p < 0.05).

**Figure 5:**
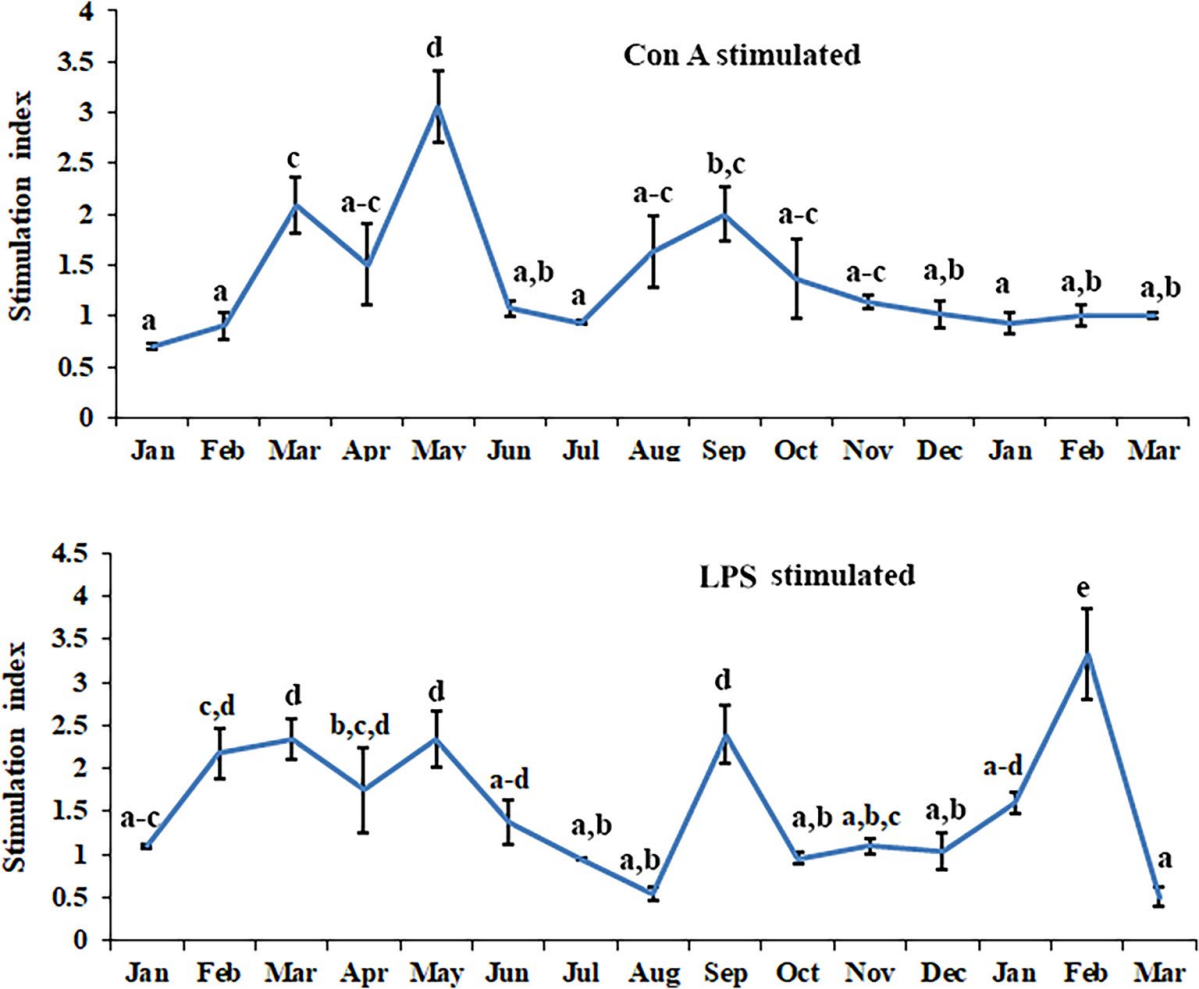
Seasonal changes in the mitogens induced lymphocyte proliferation in *N. piscator.*The error bars bearing the same superscript do not differ significantly (Newman–Keul’s multiple-range test, p < 0.05). Mitogen: *Con A* Concanavalin A, 5 µg ml^−1^, *LPS* Lipopolysaccharide, 10 µg ml^−1^.

Nitrite release by the leucocytes during different months is shown in Fig. 3. A statistically significant rhythm (df = 60; F = 35.7915 and p < 0.001) was present in nitrite release when 6 months data was Cosinor analyzed. Leucocyte phagocytosis (measured as percentage phagocytosis and phagocytic index) during different months is shown in Fig. 4. Cosinor analysis revealed that leucocyte phagocytosis is not a rhythmic phenomenon. Seasonal changes in lymphocyte proliferation are depicted in Fig. 5. Mitogen-stimulated lymphocyte proliferation also showed significant annual rhythm. The T-cell mitogen Con A-stimulated proliferation of lymphocytes was significant for the 12 months and 06 months periods (df = 60; F = 7.505; p < 0.05), but B-cell mitogen LPS-stimulated proliferation was significant for 12 months period only (df = 60; F = 9.291; p < 0.05).

## Discussion

The duration of light on the earth is the main external cue that animals utilize to determine seasonality. Animals residing in tropical regions show seasonality in their physiological processes to cope with the changing environment. For example, reproduction is an important physiological process and tropical reptiles reproduce seasonally which is an ultimate restriction to reproduction during certain times of the year^36^. Our findings suggest that reptilian immune status is a dynamic function that is dependent upon the time of the year. In the present study, the number of leucocytes was elevated during winter. In contrast, we previously found that short photoperiod suppresses leucocyte number in *N. piscator*^16^. The disparity in the results may be attributed to the differential effect of photoperiodic modulation in this species. Authors of various studies have found that blood immune cells are differentially altered after photoperiodic manipulation^37–40^. The comparative study of variation in leucocyte number demonstrates that seasonal fluctuation in the cell population is an adaptive strategy to cope with seasonal stressors^41^.

The respiratory burst activity is a crucial function of the cells involved in combating pathogens. The cells utilize reactive oxygen intermediates (ROI) as a cytotoxic substance to eliminate the pathogens. It has been postulated that in the non-mammals, the respiratory burst activity of leucocytes is compromised in winter due to cold stress^42–45^. When *N. piscator* was treated with different photoperiodic regimens for 30 days, the superoxide production did not vary significantly^16^. However, in the present work, we have found a significant annual rhythm in superoxide production by leucocytes being highest during winter. The qualitative assay (NBT slide assay) revealed that superoxide production was maximum during May. Certain species show immunological inertness as immune status does not differ after photoperiodic manipulation^46,47^. Short-day induced elevation in superoxide production can be explained by the involvement of elevated melatonin during winter^48,49^. Bowden et al.^1^ have documented that many factors are involved in altering physiological functions in *Hippoglossus hippoglossus*. Along with the seasonal changes, internal factors are also associated with the differential activity of immune cells.

Circannual rhythm in nitrite release by fish leucocytes has been demonstrated in *Channa punctatus* where authors found elevated nitrite production during spring^41,50^. Winter-induced increase in nitrite release has been reported in snake *N. piscator*^16^. In contrast to these studies, we did not find a significant annual rhythm in nitric oxide production by leucocytes in *N. piscator*. The leucocyte phagocytosis is an important aspect of the innate immune combat mechanism. The chief cells involved in phagocytosis are neutrophils and monocytes. Our study revealed the month-wise variation in phagocytic response with the highest being in autumn. This finding is similar to the findings of Munoz and Fuente^25^ who obtained the maximum adherence index of turtle leucocytes in autumn. In fish, contradictory results are available where the phagocytic response has either been elevated in summer^3,4, 51^ or another season^2,28^. The cell-mediated immune response was judged by proliferative responses of blood lymphocytes after mitogenic stimulation. The results suggested that reptilian lymphocytes show annual rhythm in proliferative response. The photoperiod is a pivotal external zeitgeber that determines the animal’s physiological response. Maintenance of snakes in altered photoperiods revealed changes in proliferative responses where lymphocyte proliferation was enhanced in cultures obtained from the animals kept either on short or long days^16^. In contrast, Kliger et al.^13^ reported that birds’ lymphocytes were non-responsive to different lighting conditions.

The finding of the annual rhythm in various immune parameters suggests that seasonality allows the animals to remain protected from seasonal stressors for a longer period. It has been stated that the energetic budget required for sustaining elevated immune status and reproduction is simultaneously incompatible^52–54^, the adaptive strategy of the animal has favored the investment of energy in these processes during a particular time of the year^55^. The possible involvement of pineal hormone cannot be ruled out which is known to be seasonally influenced and it seems feasible that during winter, when melatonin is high, the immune function is favored while reproduction is suppressed. The energy trade-off hypothesis has been proposed in mammals where animal partitions the energy among different physiological processes to obtain elevated reproductive success given the constraints of seasonal infections^56^. On the contrary, in reptiles, such energy trade-off mechanisms between reproduction and immunity are not the same as those of mammals. Earlier studies have proved that during the limitation of resources only, the immune-suppression state occurs which specifies that the earlier mentioned hypothesis is not an obligatory phenomenon but rather a facultative action^57^. Annual fluctuations in the immune status of an organism protect against the abiotic and biotic seasonal stressors which could otherwise compromise organism’s survival. *N. piscator* is a subtropical seasonal breeder and in seasonal breeders, physiological processes are rhythmic phenomena. Most physiological processes are energetically costly, therefore, life characteristics, that distinguish subtropical and temperate animals, play an important role in maintaining energetic budget. There is no known example where all physiological responses (for example immunity and reproduction) are optimum^52–54^, animals have evolved a strategy to show the optimum response for a particular physiological response at a certain time of year. One opinion suggests that when climatic conditions are unfavorable for reproduction, immunity is elevated^16^. Annual rhythm in immune function seems to be associated with the rhythmicity in the prevalence of diseases. The authors of various studies have reported the annual cycle of diseases^58,59^, it is plausible to state that annual rhythmicity in immune functions of seasonal breeders helps them fight the annual pathogenic challenges. It was concluded that seasonality in immunity is a differential adaptive functional response where immune responses are suppressed at certain times of the year when environmental conditions are most suited for reproduction. This study also provides evidence that the immune system of reptiles undergoes seasonal variation and understanding this phenomenon is crucial for comprehending the adaptive nature of immunity and its responsiveness to environmental factors. The possible reasons for many annual rhythms in immune functions are still unexplained. The molecular mechanisms underlying the annual variations in investigated immune parameters are the limitations of our studies. More studies are required to investigate the annual rhythmicity in the immune-related genes.

## Materials and methods

### Animals

Male checkered keelback *Natrix piscator* weighing about 100–120 g was procured locally from the small ponds and pits in Varanasi (28° 18′ N; 83° 01′ E), India during the start of each month. The animals were brought to the laboratory experiencing natural day length and were acclimated for 2 weeks. The animals were housed in 50 × 30 × 30 cm wooden cages with wire mesh sides accommodating five snakes per cage. An earthen vessel of 4 L was filled with water and kept in each cage and the animals were provided with small fish as food ab libitum. To maintain hygiene, cages were cleaned daily and water in the earthen vessel was replaced. The regulations of the Committee for Control and Supervision of Experiments on Animals (CPCSEA), Ministry of Statistics & Programme Implementation, Government of India, were followed strictly in the housing of the animals. The experimental protocols were approved by the Udai Pratap Autonomous College Ethical Committee. The ARRIVE and local guidelines were strictly followed during the experiment.

### Chemicals

Culture medium (RPMI-1640), lymphocyte separation medium (HiSep), fetal bovine serum (FBS), gentamycin, dimethyl sulfoxide (DMSO), l-glutamine, and other chemicals were obtained from Himedia Lab Pvt. Ltd., India. MTT [3-(4, 5-dimethylthiozol-2-yl)-2, 5 diphenyl tetrazolium bromide, NBT (Nitroblue Tetrazolium salt), melatonin, and mitogens (Con A, Concanavalin A; LPS, Lipopolysaccharide) were purchased from Sigma Chemicals, USA. The culture medium was supplemented with 1 µL ml^−1^ Gentamycin, 10 µl ml^−1^ of 200 mM l-glutamine, 10 µl ml^−1^ antibiotic–antimycotic (Gibco) and 5% FBS and was termed a complete culture medium.

### Experimental design

The experiment was carried out for a total of fifteen months and five different animals were mildly anesthetized using sodium pentobarbital at a concentration of 20 mg/kg body weight during mid of each month. In this way, a total of 75 individuals were used. The blood (about 2 ml) from each individual was isolated through cardiocentesis in heparinized tubes at 10.00 h using a 30 G-needle. Only large adult male snakes (> 100 g) were used for sampling to avoid the possible detrimental effects of the procedure on smaller snakes. The blood was employed to study total and differential leucocyte count. The leucocytes were segregated from the whole blood and used to study leucocyte phagocytosis, NBT (Nitroblue Tetrazolium) slide assay, quantitative NBT reduction assay, and nitrite assay. Lymphocytes from different experimental snakes were also incubated with B- and T-cell mitogens to evaluate the proliferative responses.

### Total leucocyte count (TLC)

Leucocytes were counted using Turk’s stain (0.2% gentian violet in 3% glacial acetic acid)^60^. Twenty microliters of blood was mixed with 380 µl of stain. The blood was then smeared on Hemocytometer and leucocytes from the four corner chambers were counted.

### Differential leucocyte count (DLC)

A thin blood smear was prepared on a clean glass slide, air dried, fixed in methanol^61^ and stained in the mixture of Leishman and Giemsa stain^62,63^. Excess stain was removed by washing the slides in running water. After washing the slides were air dried, dehydrated, cleaned in xylene and mounted in DPX. The stained slides were observed under the microscope (magnification 1000×) and 100 leucocytes from each individual were identified based on their notable morphology and counted from different areas of the slide. The percentages of different leucocytes were converted into /mm^3^ from the total leucocyte count.

### Leucocyte phagocytic assay

The yeast cells were used as the target to study leucocyte phagocytosis. Commercial baker’s yeast (*Saccharomyces cerevisae*) (20 mg) was mixed in 0.2 M PBS (10 ml) and the cells were heat killed at 80 °C. The yeast cell suspension was then centrifuged and washed in PBS. The cells were finally mixed in culture medium. The cells were counted using hemocytometer and adjusted to 1 × 10^8^ cells ml^−1^. The method of Soltanian^64^ was followed with slight modifications. Briefly, in a centrifuge tube, 20 µl of blood was mixed with 20 µl of yeast cell suspension and the mixture was incubated for 30 min, after which, a thin smear was formed on a clean glass slide. The slide was air-dried, fixed in methanol, stained with Giemsa, and examined under a microscope. Percentage phagocytosis and phagocytic index were calculated by counting 100 cells from each slide. The percentage phagocytosis was obtained by dividing the number of leucocytes showing phagocytosis by 100. The phagocytic index was obtained by counting the average number of yeast cells phagocytosed by a single leucocyte.

### Segregation of leucocytes from whole blood

Blood leucocytes were separated from the layer of leucocytes between the plasma and RBCs (buffy coat) by a slow spinning procedure as developed by Keller et al.^65^. A swinging bucket rotor was used to spin the blood at 42 × *g* for 25 min. The leucocytes were segregated by spinning the buffy and transferring the leucocytes into a new tube. The suspension was again centrifuged at 200 × *g* for 10 min, the plasma was rejected and the pellet was suspended in the culture medium. To eliminate any residual plasma, the cells were centrifuged again at 200 × *g* for 10 min, the supernatant was rejected, and the cells were resuspended in the culture medium. The number, purity and viability of leucocytes were assessed by performing the trypan blue exclusion test. The experiment was further processed only when viability exceeded 90%.

### Nitroblue tetrazolium assay (NBT assay)

The respiratory burst function and superoxide anion production by leucocytes was examined through NBT assay^66,67^. NBT is a dye that is permeable to the membrane and is reduced to purple-colored NBT-diformazan by superoxide. NBT assay is a quantitative method to determine the respiratory burst function of the cell and this technique is validated in non-mammals^68^. The reduction assay method of Berger and Slapnickova^69^ was followed to perform the NBT assay. In short, 0.1% of NBT solution was prepared in RPMI and was kept at 4 °C. Segregated leucocytes were counted and maintained at 2 × 10^6^ cells ml^−1^ in RPMI. Leucocyte viability, as determined by the trypan blue exclusion test, exceeded 95%. Leucocyte suspension (50 µl) having 1 × 10^5^ cells was mixed with 50 µl of culture medium containing NBT in the culture plate. NBT assay was done in triplicate from each animal. RPMI (100 ml) alone in triplicates served as blank. The suspension was then incubated in CO_2_ atmosphere (5%) at 25 °C for 2 h, centrifuged at 700xg, washed thrice with PBS and fixed in 70% methanol. Further, 20 µl of triton X-100 (0.1%) was added to each culture well. To dissolve the formazan crystals inside the cells 120 µl KOH (2 M) and 140 µl DMSO were added to each well. Absorbance was measured with the help of ELISA plate reader at 620 nm.

### Nitrite assay

Immune cell cytotoxicity is a crucial method to kill the invading pathogen and involves various effector molecules such as nitric oxide (NO). The literature survey has revealed that phagocytes in reptiles produce NO, which has been implicated in cytotoxic activity^70^. Nitric oxide is very unstable and is degraded to other more stable intermediates such as nitrite (NO_2_^–^) and nitrate (NO_3_^–^), popularly called reactive nitrogen intermediate (RNI). The concentration of nitrite intermediates in the supernatant was determined following the method of Ding et al.^71^ with slight changes. Leucocyte suspension (100 µl) containing 1 × 10^6^ cells ml^−1^ was placed in the well of 96 well culture plate. Leucocytes were incubated at 25 °C in CO_2_ atmosphere (5%) for 24 h, centrifuged at 200 × g and the supernatant was aspirated in another well. An equal volume of Griess reagent (1% sulfanilamide in 3N HCl and 0.1% naphthylene diamine dihydrochloride in distilled water) and supernatant were mixed and absorbance of the solution was read at 540 nm with the help of microplate reader. Wells having culture medium alone without any cell served as blank.

### Lymphocyte proliferation assay

The proliferation of lymphocytes was employed to evaluate the cell-mediated immune response. The proliferation assay was performed using a colorimetric assay which involves the use of tetrazolium salt (MTT) as described by Berridge et al.^72^. Tetrazolium salts cross the plasma membrane and enter the mitochondria of metabolically active leucocytes. The mitochondrial enzyme cleaves the tetrazolium rings of MTT which is then bio-reduced into dark blue formazan crystals which remain inside the cells. The addition of a detergent into the culture solubilizes the cell and the formazan crystals are released. MTT assay provides a measure of cell number during the last moments of in vitro culture. Lymphocytes were separated from the blood by centrifugation (density gradient) using HiSep having density of 1.077 g ml^−1^. Blood was carefully overlaid on an equal volume of HiSep in a test tube and centrifuged at 400 × g for 30 min at 8 °C with brakes off. After centrifugation, a ring of lymphocyte formed at the interface between medium and HiSep was carefully taken out, washed thrice with PBS, counted and viability was checked through trypan blue exclusion test. The viable cells that exceeded 95% were adjusted to 2 × 10^6^ cells ml^−1^ in culture medium.

Basal and mitogen (Con A and LPS) activated cell proliferation assays were done. Mitogen’s stock solutions were made in 0.2 M PBS (pH 7.2) at a concentration of 1 mg ml^−1^ and further working dilutions were prepared in culture medium. Con A was used at concentration of 10 µg ml^−1^ while LPS was used at concentration of 20 µg ml^−1^. Fifty micro liters of mitogen and 50 µl of cell suspension, having 2 × 10^6^ cells ml^−1^, were taken in the wells of the culture plate. To study basal proliferation, 1 × 10^5^ cells were added into the well of the culture plate along with 50 µl of culture medium. Wells containing only 100 µl of culture medium acted as blank. Plates were incubated in humidified 5% CO_2_ atmosphere for 48 h at 25 °C. After incubation, 10 µl of MTT reagent (5 mg ml^−1^) was added to each well, and plates were incubated for 4 h. Following incubation, plates were centrifuged at 200 × g and the supernatant was aspirated. To solubilize the formazan crystals 100 µl of DMSO was added to each well and optical density was read at 570 nm with the help of an ELISA plate reader.

### Statistical analysis

The data were processed by one-way Analysis of Variance (ANOVA) using SPSS and Post hoc comparisons were made using Newman-Keul’s multiple-range test. The significance of the difference was considered when P < 0.05. The seasonal data were also evaluated by the Cosinor analysis to validate the annual rhythm. Cosinor analysis is a popular tool for analyzing the biological time series with discernible rhythms using the least square method to implement sine wave in the provided time series. The Cosinor rhythmometry analysis is the conventional method to analyze circannual rhythm which determines the degree of fit between the user-defined model consisting of a supra-position of cosine functions and the experimental data and model. The annual rhythm was characterized by various parameters such as mesor (rhythm-adjusted mean), amplitude (A), acrophase (ϕ), and p indicating the significance of the rhythm.

## Data Availability

All the data pertaining to the findings are available in the manuscript in the form of graphs and tables. Any further query may be addressed to the corresponding author.
